# Giant Elastocaloric Effect and Improved Cyclic Stability in a Directionally Solidified (Ni_50_Mn_31_Ti_19_)_99_B_1_ Alloy

**DOI:** 10.3390/ma17194756

**Published:** 2024-09-27

**Authors:** Honglin Wang, Yueping Wang, Guoyao Zhang, Zongbin Li, Jiajing Yang, Jinwei Li, Bo Yang, Haile Yan, Liang Zuo

**Affiliations:** 1 Key Laboratory for Anisotropy and Texture of Materials (Ministry of Education), School of Materials Science and Engineering, Northeastern University, Shenyang 110819, China; homlee_wang@163.com (H.W.); ysuwangyueping@163.com (Y.W.); zhanggy_neu@foxmail.com (G.Z.); yangb@atm.neu.edu.cn (B.Y.); yanhaile@mail.neu.edu.cn (H.Y.); lzuo@mail.neu.edu.cn (L.Z.); 2 Western Metal Materials Co., Ltd., Xi’an 710201, China; yjj02030222@163.com; 3 Liaoning Automobile Lightweight Professional Technology Innovation Center, Tieling 112000, China; lijinwei_ln@126.com

**Keywords:** elastocaloric effect, Ni-Mn-Ti alloys, stress-induced martensitic transformation, cyclic stability

## Abstract

Superelastic shape memory alloys with an integration of large elastocaloric response and good cyclability are crucially demanded for the advancement of solid-state elastocaloric cooling technology. In this study, we demonstrate a giant elastocaloric effect with improved cyclic stability in a <001>_A_ textured polycrystalline (Ni_50_Mn_31_Ti_19_)_99_B_1_ alloy developed through directional solidification. It is shown that large adiabatic temperature variation (|Δ*T_ad_*|) values more than 15 K are obtained across the temperature range from 283 K to 373 K. In particular, a giant Δ*T_ad_* up to −27.2 K is achieved by unloading from a relatively low compressive stress of 412 MPa at 303 K. Moreover, persistent |Δ*T_ad_*| values exceeding 8.5 K are sustained for over 12,000 cycles, exhibiting a very low attenuation behavior with a rate of 7.5 × 10^−5^ K per cycle. The enhanced elastocaloric properties observed in the present alloy are ascribed to the microstructure texturing as well as the introduction of a secondary phase due to boron alloying.

## 1. Introduction

Refrigeration systems such as air-conditioners and refrigerators are playing an increasingly important role in modern life. Vapor compression remains the predominant technology in refrigeration facilities, yet the high global-warming potential of volatile liquid refrigerants has raised extensive concerns on environmental degradation [1]. Elastocaloric refrigeration, which is based on the elastocaloric effect (eCE) of solid-state materials, offers an eco-friendly cooling alternative that circumvents potential impacts on the global environment [2,3,4]. Shape-memory alloys (SMAs) show great potential as candidate materials for elastocaloric cooling due to their ability to exhibit an elastocaloric response through the utilization of released and absorbed latent heat in association with stress-induced martensitic transformation. Consequently, very pronounced isothermal entropy change (Δ*S_iso_*) or adiabatic temperature change (Δ*T_ad_*) can be expected upon the application or removal of certain uniaxial stresses [5].

Heusler-type Ni-Mn-X (e.g., X = Ga, In, Sn)-based shape-memory alloys have been shown to exhibit various functional behaviors by utilizing the martensitic transformation, such as magnetic shape memory effect [6,7], magnetocaloric effect [8,9], and elastocaloric effect [10]. However, the cycling life of elastocaloric response in these alloys suffers from strong constraint of poor mechanical properties (e.g., 15 cycles for a Ni_50_Mn_33_In_15.5_Cu_1.5_ alloy [11] and 100 cycles for a Ni_50.4_Mn_27.3_Ga_22.3_ alloy [12]), making them difficult to become reliable elastocaloric refrigerants [10,11,12,13]. The recently developed all-*d*-metal Heusler-type Ni-Mn-Ti alloys have attracted considerable attention owing to the enhanced mechanical properties that are achieved by the hybridization of *d*-*d* atomic orbitals [14]. These alloys exhibit great potential in cooling applications owing to the very large volume change in unit cell (Δ*V*/*V_0_*) between austenite and martensite, which substantially augments the entropy change Δ*S_tr_* of martensitic transformation and consequently results in very prominent elastocaloric response [15]. In a polycrystalline (Ni_50_Mn_31.5_Ti_18.5_)_99.8_B_0.2_ alloy, it has been reported that removing a compressive loading of 700 MPa can yield a colossal adiabatic temperature drop of up to −31.5 K [15].

Although Ni-Mn-Ti alloys exhibit improved mechanical properties and elastocaloric effect when compared to some other typical Ni-Mn-X-based alloys [10,11,16], there remains a lack of sufficient cyclability in polycrystalline alloys, e.g., fewer than 100 cycles in a Ni_50_Mn_31.5_Ti_18_Cu_0.5_ alloy [17]. The primary cause of this dilemma stems from the constraining effect of adjacent grain boundaries in conventional polycrystalline alloys since the stress-induced martensitic transformation is very sensitive to crystallographic orientation, resulting in deformation incompatibility among grains with different crystallographic orientations. Consequently, significant stress concentration occurs at triple junctions with increasing superelastic cycles, ultimately leading to sample fracture. To enhance the deformation compatibility of neighboring grains during phase transformation, an alternative strategy is to develop a highly textured microstructure with columnar grains through directional solidification [11,18]. Moreover, it is noted that the necessary driving stress for achieving a colossal elastocaloric effect in polycrystalline Ni-Mn-Ti alloys remains too high [14,19]. Since the transformation stress can be effectively manipulated by crystallographic orientation [20,21], microstructure texturing is also expected to reduce the driving stress of martensitic transformation. In addition, alloying new elements is known to be very effective in tailoring the mechanical properties and functional stability [22,23]. Recently, the cyclic number for a (Ni_50_Mn_30.75_Ti_18.25_Cu_1_)_99.8_B_0.2_ polycrystalline alloy has been successfully increased to 650 cycles [24], owing to the substantial increase in grain boundary strength due to boron doping. It is known that the precipitate phases can also be introduced by boron doping [25], which could be exploited for the improvements in mechanical properties and functional stability.

To tackle the tough challenges of relatively low cyclability and high driving stress for the elastocaloric effect in polycrystalline Ni-Mn-Ti alloys, a strategy with the combination of microstructure texturing and boron doping was employed in this work. Considering that boron doping may increase the transformation temperatures [15], a Ni_50_Mn_31_Ti_19_ alloy with relatively low transformation temperature (i.e., *M_s_* = 220 K, *M_f_* = 210 K, *A_s_* = 236 K, and *A_f_* = 243 K) was used as the starting alloy to facilitate the exploration of elastocaloric response around room temperature for the boron doped alloys. For the purpose of strengthening the matrix by utilizing the precipitates, 1% boron was introduced and a <001>_A_ textured polycrystalline (Ni_50_Mn_31_Ti_19_)_99_B_1_ alloy with a coarse columnar-grained microstructure was developed by directional solidification. Large |Δ*T_ad_*| values over 15 K were achieved when removing the compressive loading from 283 K to 373 K. Notably, an impressive Δ*T_ad_* of −27.2 K was observed at 303 K upon eliminating a relatively low compressive stress of 412 MPa. Moreover, |Δ*T_ad_*| values exceeding 8.5 K were sustained for over 12,000 cycles, showing improved cyclability owing to the synergistic effects of microstructure texturing and boron doping.

## 2. Materials and Methods

Polycrystalline (Ni_50_Mn_31_Ti_19_)_99_B_1_ (at. %) alloy was fabricated by repeatedly melting high-purity elements in a conventional arc-melting furnace. To obtain a well-oriented microstructure, directional solidification was performed by using the Bridgman method with a temperature gradient of 120 K cm^−1^ [26], where liquid Ga-In-Sn metal was utilized as the cooling medium. The arc-melted alloy was enveloped in a corundum crucible with an inner diameter of 10 mm and a length of 150 mm, and subsequently remelted at 1563 K. Following a holding time of 1 h, the alloy was directionally solidified by drawing the crucible into a cylinder filled with liquid Ga-In-Sn metal at a constant speed of 3 mm min^−1^. To eliminate the compositional inhomogeneity, the as-cast alloys were enclosed in vacuum quartz tubes and then subjected to a heat treatment at 1223 K for 48 h. Subsequently, the alloys were rapidly cooled by quenching in cold water. The composition analyses were performed using an energy dispersive spectrometer (EDS). The start and finish temperatures for the forward and reverse martensitic transformation (*M_s_*, *M_f_*, *A_s_*, *A_f_*) were determined through differential scanning calorimetry (DSC-25, TA Instruments, New Castle, USA), using a temperature scanning rate of 10 K min^−1^. The microstructural features and preferred orientation were characterized using a scanning electron microscope (SEM, JEOL, JSM-IT800, Tokyo, Japan) and the attached electron backscatter diffraction (EBSD) system. To test the mechanical properties, uniaxial compression along the solidification direction (SD) was conducted using a material testing machine with a temperature control unit (AGS-X/50 kN, Shimadzu, Kyoto, Japan). The compressive axis was aligned parallel to the longer edge of a cuboid-shaped sample sized 6 mm × 4 mm × 3 mm. A K-type thermocouple with a resolution of 0.1 K was adhered to the sample surface to track the temperature variation Δ*T_ad_* caused by rapid loading or unloading. The temperature of the sample was continuously recorded with an interval of 0.1 s. A 15 s dwell time was set between loading and unloading to ensure the thermal equilibrium between the sample and the ambience.

## 3. Results

**Figure 1:**
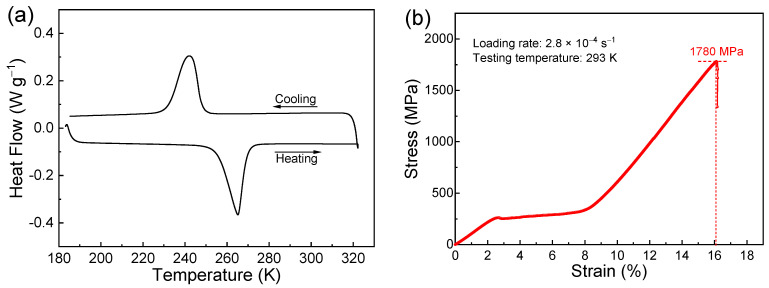
(**a**) DSC curves for the directionally solidified (Ni_50_Mn_31_Ti_19_)_99_B_1_ alloy; (**b**) compressive stress–strain curve measured at 293 K.

DSC measurements demonstrate that the martensitic transformation for the directionally solidified (Ni_50_Mn_31_Ti_19_)_99_B_1_ alloy occurs below room temperature (Figure 1a) and the corresponding characteristic temperatures are determined to be *M_s_* = 248 K, *M_f_* = 232 K, *A_s_* = 255 K, and *A_f_* = 269 K. In addition, the entropy change Δ*S_tr_* related to martensitic transformation can be evaluated to be 66.7 Jkg^−1^K^−1^. Figure 1b plots the compressive stress–strain relation tested at 293 K with a strain rate of 2.8 × 10^−4^ s^−1^. The directionally solidified alloy can withstand a maximum compressive strength of ~1780 MPa, which is much higher than that of the arc-melted alloy (i.e., 1460 MPa, Supplementary Figure S1). In addition, the compressive strength for the present directionally solidified alloy is also higher than those of some other polycrystalline alloys, such as Ni_50_Mn_31.6_Ti_18.4_ alloy (i.e., 800 MPa) [27], Ni_50_Mn_31.75_Ti_18.25_ alloy (i.e., 1100 MPa) [14], and Ni_36.5_Co_13.5_Mn_35_Ti_14.1_Gd_0.9_ alloy (i.e., 1142 MPa) [28]. Achieving enhanced mechanical properties is of great significance for the functional stability of elastocaloric materials [29].

**Figure 2:**
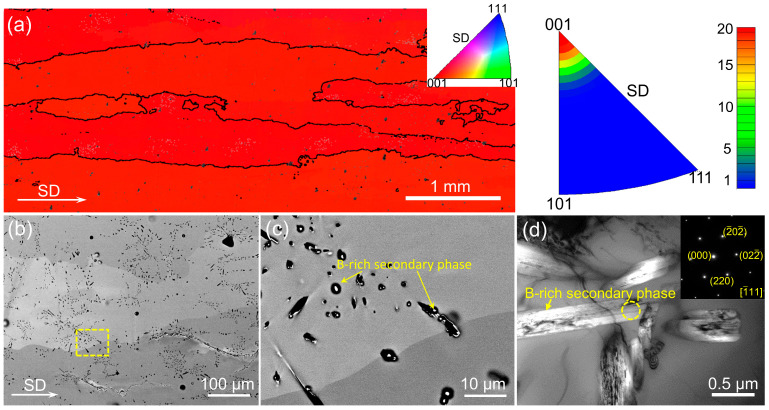
(**a**) EBSD orientation micrograph (IPF contrast) covering the longitudinal section for the (Ni_50_Mn_31_Ti_19_)_99_B_1_ alloy and the corresponding inverse pole figure related to the SD; (**b**) backscattered electron (BSE) image for the directionally solidified alloy; (**c**) BSE image corresponding to the squared region of (**b**); (**d**) TEM bright field image and the corresponding selected-area electron diffraction (SAED) for the boron-rich secondary phase along the [$\overset{-}{1}$11] axis.

The global microstructure of the directionally solidified (Ni_50_Mn_31_Ti_19_)_99_B_1_ alloy is characterized by large columnar grains, with their longer axes along the SD. Figure 2a displays an EBSD orientation map colored in IPF contrast, covering the region of 5.5 mm × 2.2 mm. It is seen that the columnar grains have a homogeneous orientation with <001>_A_ along the SD, i.e., <001>_A_ fiber texture along the SD. As demonstrated in the corresponding inverse pole figure, the texture strength of <001>_A_ fiber texture is 19.6. The formation of such highly textured microstructure should be attributed to the preferential growth of <001>_A_ along the temperature gradient direction [30]. In contrast, the arc-melted alloy exhibits equiaxed grains with a very low degree of preferred orientation (Supplementary Figure S2). Thus, it can be inferred that the enhanced mechanical properties of the directionally solidified alloy should be mainly attributed to the columnar-grained microstructure with a strong <001>_A_ preferred orientation. Such a specific microstructural feature contributes to enhancing the mechanical properties by minimizing stress concentrations at triple junctions and promoting better strain compatibility among adjacent grains. [31]. Microstructural observations also show that certain amounts of precipitated phase appear in the studied alloy, as shown in Figure 2b,c. As determined from the EDS measurements, the matrix has an average composition of Ni_50.3_Mn_30.9_Ti_18.8_, being close to the nominal composition, and the precipitates correspond to a boron-rich phase [32]. Furthermore, a boron-rich secondary phase is observed by TEM, as shown in Figure 2d. It can be observed that the secondary phase is in rod-like shape. The corresponding SAED indicates that the second phase exhibits a face-centered cubic structure (inset of Figure 2d). Similar observations of boron-rich precipitates have also been demonstrated in other boron-doped Ni-Mn-based alloys [13]. The introduction of precipitates has a positive effect in inhibiting crack propagation and alleviating extensive stress concentration [13,24,33,34]. Thus, the enhanced mechanical properties in the present alloy should result from the highly textured microstructure and the introduction of secondary phase due to boron alloying [35,36].

**Figure 3:**
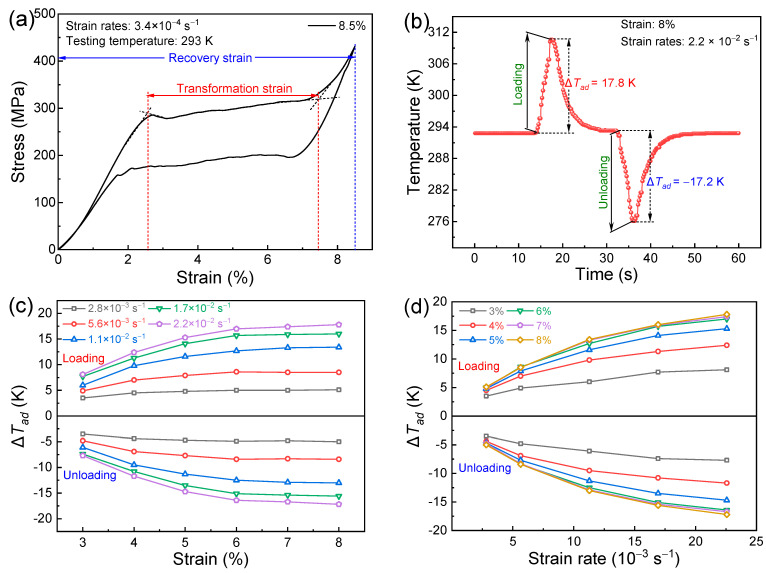
(**a**) Superelastic loop of the directionally solidified alloy measured under compression at 293 K; (**b**) typical Δ*T_ad_* profile under a compressive strain of 8% by applying and removing the compressive loading of 510 MPa at 293 K; (**c**) correlation between Δ*T_ad_* and compressive strain under various strain rates tested at 293 K; (**d**) correlation between Δ*T_ad_* and strain rate under various compressive strains tested at 293 K.

Prior to the characterizations on the superelastic and elastocaloric properties, mechanical training was conducted by performing several cycles of loading–unloading to stabilize stress-induced martensitic transformation (Supplementary Figure S3). Figure 3a presents the superelastic loop for the directionally solidified (Ni_50_Mn_31_Ti_19_)_99_B_1_ alloy tested at 293 K. Typical plateau-like superelastic behavior with a stress hysteresis of 105 MPa can be clearly observed. Because of the well-developed <001>_A_ preferential orientation, a large transformation strain of 4.9% is obtained by stress-induced transformation, enabling a recoverable strain of 8.5% upon unloading.

**Figure 4:**
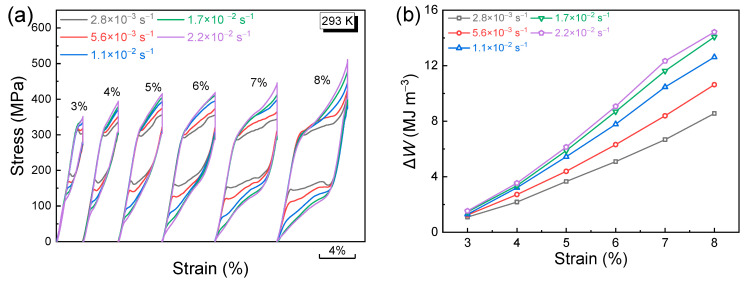
(**a**) Influence of strain rate on the superelastic response at various compressive strains tested at 293 K, by applying the maximum compressive loading of 350 MPa, 394 MPa, 415 MPa, 417 MPa, 445 MPa, and 506 MPa for the compressive strains of 3%, 4%, 5%, 6%, 7%, and 8%, respectively; (**b**) correlation between energy dissipation Δ*W* and compressive strain under various strain rates.

In order to analyze the elastocaloric properties, the Δ*T_ad_* values obtained by applying/releasing the compressive stress were directly measured. A typical Δ*T_ad_* profile is displayed in Figure 3b, showing an adiabatic temperature rise on loading and a temperature drop upon unloading. Figure 3c depicts the variation in Δ*T_ad_* values with compressive strains detected under various strain rates. The |Δ*T_ad_*| values increase with increasing compressive strain owing to the gradually enhanced proportion of transformed martensite. A rapid increase in |Δ*T_ad_*| values is presented until the compressive strain of 7% and then the increasing rate becomes slow. When applying a compressive strain of 8%, the Δ*T_ad_* values resulting from the application and release of compressive stress at a strain rate of 2.2 × 10^−2^ s^−1^ reach 17.8 K and −17.2 K, respectively. Figure 3d presents the correlation between Δ*T_ad_* and strain rate under various compressive strains. Apparently, increasing the strain rate results in a gradual rise in |Δ*T_ad_*| values on account of the improved adiabatic condition. Thus, both the applied strain and strain rate strongly affect the stress-induced Δ*T_ad_* values. The corresponding stress–strain curves for Δ*T_ad_* measurements under various compressive strains and strain rates are displayed in Figure 4a.

**Figure 5:**
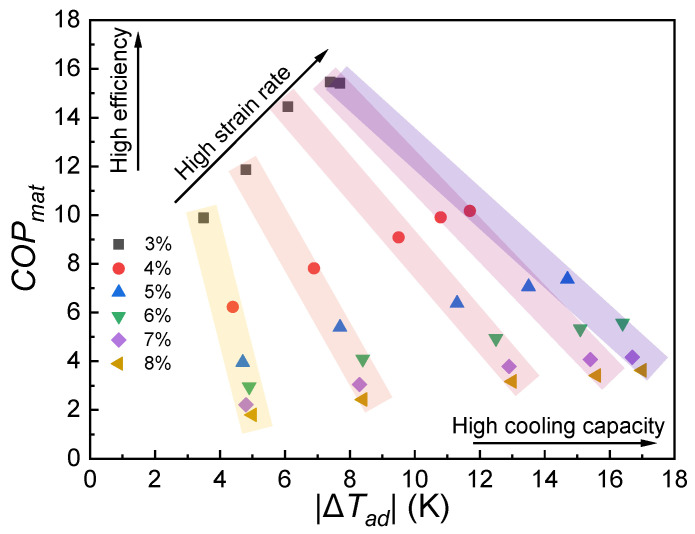
Graphical representation of *COP_mat_* as a function of Δ*T_ad_*.

It is noted that applying a higher compressive stain and strain rate also widens the stress hysteresis, resulting in enhanced energy dissipation (Δ*W*) that is represented by the enclosed region of the superelastic loop, as demonstrated in Figure 4. Such energy dissipation is irreversible, thereby weakening the energy conversion efficiency [37]. An important index used for evaluating the energy efficiency is the coefficient of performance of material (*COP*_mat_), which can be calculated by Equation (1) [38].

(1) $${\mathrm{COP}}_{\mathrm{mat}}=|\Delta Q/\Delta W|=|(\rho \Delta T_{\mathrm{ad}}\cdot C_{p})/\oint \sigma d\varepsilon |$$

where *ρ* denotes the density (*ρ* = 7278 kg m^−3^) and *C_p_* is the specific heat capacity (*C_p_* = 423 Jkg^−1^K^−1^, Supplementary Figure S4). Based on the Δ*T_ad_* and Δ*W* mentioned above, the influence of strain level and strain rate on the *COP*_mat_ was analyzed, as summarized in Figure 5. Generally, the *COP*_mat_ values are gradually increased with the strain rate at a constant compressive strain. However, when the strain rate is fixed, the *COP*_mat_ values are significantly reduced when increasing the compressive strain, even though there is a significant improvement in Δ*T_ad_*.

**Figure 6:**
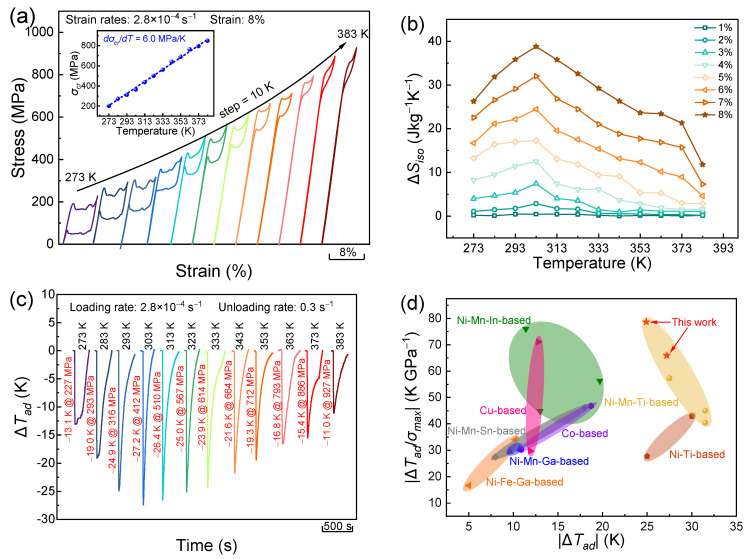
(**a**) Temperature-dependent superelasticity of the present alloy subjected to a strain rate of 2.8 × 10^−4^ s^−1^ by applying a maximum compressive strain of 8%. The inset displays the evolution of critical stress *σ_cr_* as the variation of temperature. (**b**) Δ*S_iso_* induced by compressive loading under various compressive strains. (**c**) Δ*T_ad_* profiles upon unloading measured at various temperatures. (**d**) Comparison of the elastocaloric properties between the present alloy and several typical elastocaloric materials [3,10,11,15,16,20,31,39,40,49,50,51,52,53,54,55,56].

In order to examine the influence of testing temperature on the superelastic response, compressive tests were conducted over a temperature range from 273 K to 383 K, by using a compressive strain of 8% at a strain rate of 2.8 × 10^−4^, as shown in Figure 6a. At each testing temperature, the alloy demonstrates a typical plateau-type superelasticity and a reversible strain of 8% can be clearly observed. With the increase in testing temperature, a higher level of driving stress is needed to activate the martensitic transformation owing to the increased stability of austenite. Consequently, the critical stress σ_cr_ shows a linearly increasing trend, with a rate of 6.0 MPa K^−1^ (inset of Figure 6a). Similar linear dependence has also been reported in some other Ni-Mn-Ti alloys [15,39]. According to the stress–strain curves obtained at different temperatures, the compressive loading-induced Δ*S_iso_* was evaluated through the Maxwell relation (Equation (2)).

(2) $$\Delta S_{\mathrm{iso}}={\;v}_{0}\int_{0}^{\varepsilon }{\left(\frac{\partial \sigma }{\partial T}\right)}_{\varepsilon }d\varepsilon$$

In the equation, *v_0_* refers to the specific volume (i.e., 1.38 × 10^−4^ m^3^kg^−3^), as shown in Figure 6b. Under the compressive strain of 8%, a maximum Δ*S_iso_* of 38.8 Jkg^−1^K^−1^ can be achieved at 303 K.

The unloading Δ*T_ad_* profiles measured at the temperature span from 273 K to 383 K when removing a compressive strain of 8% are presented in Figure 6c. Here, the unloading process was performed by using a much higher strain rate of 0.3 s^−1^, thereby allowing the enhanced adiabatic condition. A giant elastocaloric response, characterized by |Δ*T_ad_*| values exceeding 15 K, was obtained within the testing temperature region from 283 K to 373 K. It is worth noting that the most remarkable elastocaloric response appears at 303 K, showing the maximum Δ*T_ad_* up to −27.2 K. Such Δ*T_ad_* is in good agreement with the value evaluated from the Δ*S_iso_* (i.e., −27.6 K) based on Equation (3).

(3) $$\Delta T_{\mathrm{ad}}^{\mathrm{cal}}=\Delta S_{\mathrm{iso}}\cdot T_{\mathrm{test}}/C_{p}$$

Table 1 compares the elastocaloric properties between the present alloy and some other elastocaloric materials. It can be seen that the |Δ*T_ad_*| value (i.e., 27.2 K) of the current alloy is higher than those of Ni-Mn-X alloys (X = Ga, In, Sn and Sb) [10,40,41,42,43,44], Cu-based alloys [45,46], and Ni-Ti alloys [46,47], but lower than those of certain Ni-Mn-Ti alloys. It should be noted that the colossal elastocaloric effect in those Ni-Mn-Ti alloys requires a quite large driving stress, indicating a high level of energy consumption. By comparison, the required external stress for the present alloy is substantially decreased, i.e., 412 MPa, thereby favoring the miniaturization and energy-saving of refrigeration equipment [48]. To quantify the elastocaloric response, the adiabatic temperature change under unit stress was determined by |Δ*T_ad_*/*σ_max_*|. Notably, the maximum |Δ*T_ad_*/*σ_max_*| of the present alloy reaches 78.8 K GPa^−1^, owing to a considerably large Δ*T_ad_* of −24.9 K realized at a relatively low compressive stress of 316 MPa at 293 K. By comparison, the present |*T_ad_*/*σ_max_*| is also larger than those of some other elastocaloric materials [3,10,11,15,16,20,31,39,40,49,50,51,52,53,54,55,56], as illustrated in Figure 6d.

**Figure 7:**
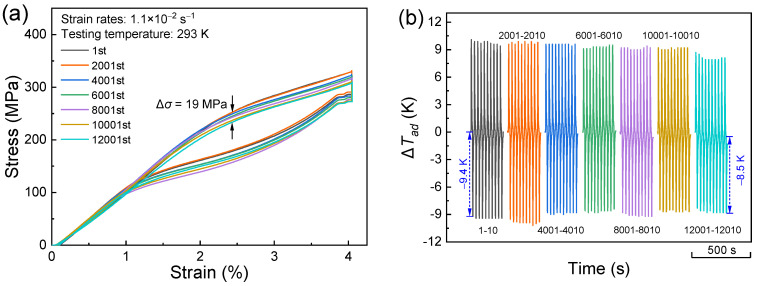
(**a**) Selective stress–strain curves during cyclic tests; (**b**) Δ*T_ad_* profiles induced by loading and unloading for some selected cycles.

To investigate the cyclability of eCE for the present alloy, cyclic tests under a compressive strain of 4% were performed by using a strain rate of 1.1 × 10^−2^ s^−1^. Figure 7a displays the evolution of stress–strain correlation during the cyclic tests. The superelastic response demonstrates a good stability, with a small stress drop of 19 MPa over 12,000 cycles of testing. Such a stress drop could be caused by the accumulation of transformation-induced defects that stabilize the martensite phase [55,58]. It is noted that there is no indication of fracture on the sample after 12,000 cycles, which is also a reflection of enhanced mechanical properties in the present directionally solidified alloys. Figure 7b presents the representative Δ*T_ad_* profiles corresponding to the various selected cycles. The present alloy exhibits persistent |Δ*T_ad_*| values larger than 8.5 K for more than 12,000 cycles. The degradation rate of |*ΔT_ad_*| values during such long-term tests is very low, i.e., 7.5 × 10^−5^ K per cycle. For a purpose of comparative analyses, the cyclic tests were also conducted in a directionally solidified Ni_50_Mn_31_Ti_19_ alloy without boron doping under the same testing parameters. As shown in Figure S5a, the stress–strain curves demonstrate a significant stress drop of 167 MPa after 40 mechanical cycles for the sample. Additionally, the |Δ*T_ad_*| values during unloading also exhibit a sharp decrease from 8.4 K to 4.1 K (Supplementary Figure S5b). Thus, boron doping indeed yields a prominent cyclability of the elastocaloric response. As summarized in Table 2, both the cycle number and degradation rate for the present alloy are substantially improved when compared to some typical elastocaloric materials reported in the literature [12,20,39,45,46,53,57,59,60,61,62,63,64,65,66,67], thereby showing an enhanced functional stability of the elastocaloric response.

**Figure 8:**
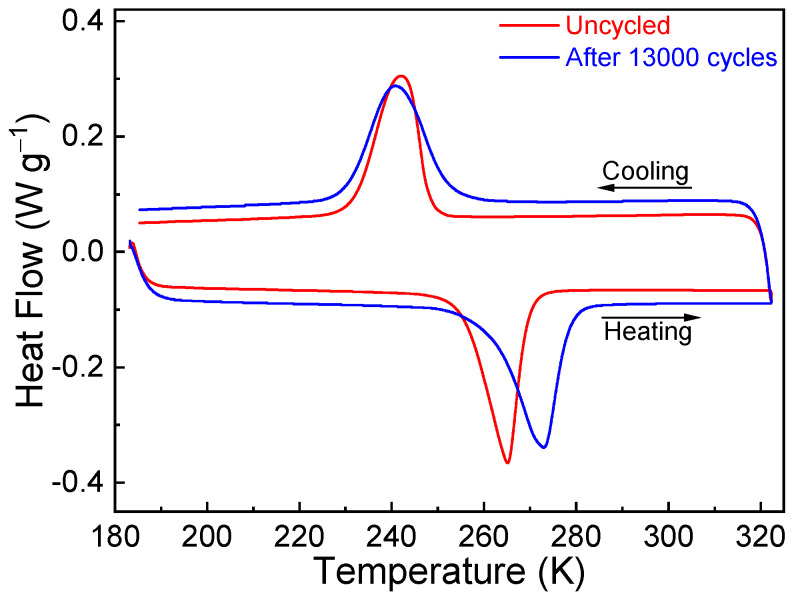
Comparison on the DSC curves for the samples before and after 12,000 mechanical cycles.

To further understand the degradation of functional behaviors, the DSC curves between the initial sample and the sample after mechanical cycles were compared, as shown in Figure 8. It is seen that mechanical cycling leads to a broadening of the endothermic/exothermic peak, which could be an indication of the accumulation of dislocations and the resulting internal stress [68]. In addition, Δ*S_tr_* is also reduced after long-term testing, i.e., from 66.7 Jkg^−1^K^−1^ for the initial sample to 59.8 Jkg^−1^K^−1^ in the cycled sample, suggesting the generation of residual martensite induced by mechanical cycling.

**Figure 9:**
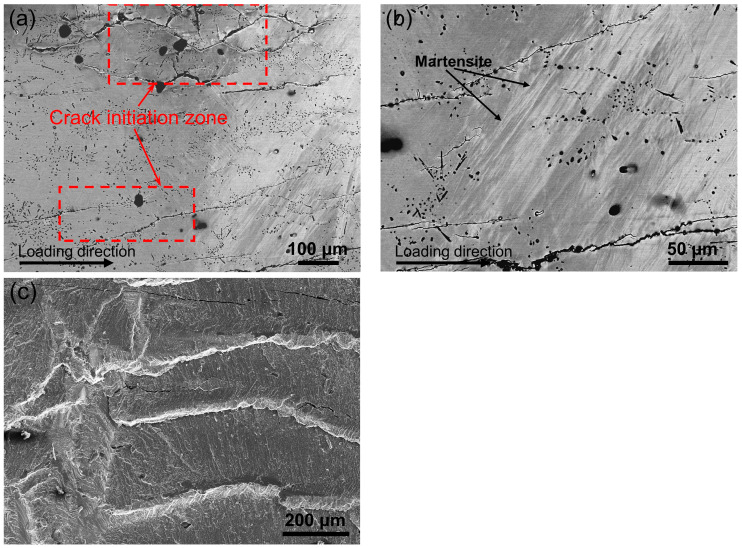
(**a**) Morphology of the longitudinal section after 12,000 mechanical cycles; (**b**) local BSE image showing the appearance of residual martensite after mechanical cycling; (**c**) fractured surface morphology after 13,000 cycles of mechanical tests.

Figure 9a shows the microstructure image for the longitudinal section of the directionally solidified (Ni_50_Mn_31_Ti_19_)_99_B_1_ alloy after 12,000 mechanical cycles. It is noted that some longitudinal cracks have nucleated and propagated along the loading direction. The occurrence of these cracks can be attributed to the generation of stress concentration at the grain boundary during mechanical cycling. With increasing the cycles, cracks nucleate at the grain boundary and subsequently propagate into the interior of the grains, as highlighted in red dashed squares in Figure 9a. It is worth mentioning that residual martensite can also observed in the alloy, as shown in Figure 9b, which should account for the reduced Δ*S_tr_* observed in the DSC measurements after mechanical cycling. Accordingly, the reduction in the volume fraction of transforming austenite involved in the phase transition results in the attenuation of Δ*T_ad_* during mechanical cycling.

To elucidate the underlying fatigue mechanisms behind long-term cyclic testing, the morphological characteristics of the fractured sample were analyzed. Figure 9c displays the morphology of the longitudinal section for the directionally solidified (Ni_50_Mn_31_Ti_19_)_99_B_1_ alloy after 13,000 mechanical cycles. The abundance of river patterns and cleavage steps suggests that the sample exhibits a transgranular fracture behavior, which aligns with the observed crack propagation observed in Figure 9a. Moreover, the cross-section exhibits numerous white granular precipitates that are dispersedly distributed along the grain boundary and within the grain. This provides clear evidence of enhanced cyclic stability by boron doping. These precipitates effectively mitigate deviatoric stress through plastic deformation, thereby impeding crack initiation and propagation [34].

The results presented above indicate that the present directionally solidified alloy exhibits a giant elastocaloric response and improved cyclic stability, which should be attributed to the effective microstructure tuning through directional solidification and boron doping. It is known that during mechanical cycling, dislocations and microcracks tend to preferentially form at fragile grain boundaries, resulting in premature intergranular fracture [69]. By utilizing directional solidification, a coarse-grained microstructure with <001>_A_ preferential orientation formed in the present alloy significantly reduces the amount of grain boundaries, thereby minimizing crack initiation sites and enabling prolonged service life [70,71]. Moreover, the well-oriented microstructure is also favorable for the improvement in the deformation compatibility during stress-induced phase transformation, which thus weakens the grain boundary constraints on the lattice deformation and contributes to a giant elastocaloric response under reduced driving stress [11]. On the other hand, boron is recognized as an electron donor [72,73], and its microalloying can effectively enhance the cohesive strength of grain boundaries, thereby increasing the mechanical properties by impeding the intergranular fracture. This effect has been observed in various structural intermetallic compounds such as Ni_3_Al [74], Ni_3_Fe [75], and FeAl [76] alloys. Here, a boron-rich secondary phase is also introduced in the present alloy due to boron doping. This secondary phase is distributed within the grain and along the grain boundary (Figure 2c), which is favorable for enhancing the mechanical properties and thus extending the service life. Overall, the enhanced elastocaloric response for the present directionally solidified alloy should primarily arise from the synergy of microstructure texturing and boron doping.

## 4. Conclusions

In summary, the elastocaloric effect and its functional stability in a <001>_A_ textured polycrystalline (Ni_50_Mn_31_Ti_19_)_99_B_1_ alloy fabricated by directional solidification were investigated. Through boron doping and microstructure control, the mechanical properties were significantly enhanced, giving rise to the maximum compressive strength of 1780 MPa. Consequently, a giant elastocaloric response represented by |Δ*T_ad_*| values higher than 15 K was realized over a wide temperature window ranging from 283 K to 373 K. Because of the reduced driving stress derived from the highly textured microstructure, a very high value of specific adiabatic temperature change (|Δ*T_ad_*/*σ_max_*|) up to 78.8 K GPa^−1^ was achieved. Moreover, persistent |Δ*T_ad_*| values exceeding 8.5 K were sustained for over 12,000 cycles, showing enhanced cyclability when compared to some other elastocaloric materials. It is demonstrated that the combination of microstructure texturing and boron doping can be exploited as an effective approach in enhancing the cyclic stability of the elastocaloric effect.

## Figures and Tables

**Table 1:** Comparison of the elastocaloric properties between the present alloy and some other elastocaloric materials.

Alloy	Sample Status	T_test_ (K)	|Δ*T_ad_*| (K)	*σ_max_* (MPa)	Strain Rate (s^−1^)	Ref.
(Ni_50_Mn_31_Ti_19_)_99_B_1_	Polycrystal (textured)	303	27.2	412	3.0 × 10^−1^	This work
Ni_50_Mn_31.75_Ti_18.25_	Polycrystal (textured)	293	20.4	913	2.0	[14]
Ni_50_Mn_35_Ti_15_	Polycrystal (untextured)	293	4.5	450	2.8 × 10^−2^	[19]
Ni_50_Mn_31.6_Ti_18.4_	Single crystal	293	29.0	830	2.0	[27]
Ni_37_Co_9_Fe_4_Mn_35_Ti_15_	Polycrystal (untextured)	293	6.3	400	1.4 × 10^−1^	[57]
(Ni_50_Mn_31.5_Ti_18.5_)_99.8_B_0.2_	Polycrystal (untextured)	308	31.5	700	5.33	[15]
Ni_55_Mn_18_Ga_27_	Polycrystal (textured)	300	10.7	350	2.0 × 10^−1^	[40]
Ni_50_Mn_18.5_Ga_25_Cu_6.5_	Polycrystal (textured)	315	8.1	100	4.2 × 10^−2^	[41]
Ni_50_(Mn_28.5_Cu_4.5_)(In_14_Ga_3_)	Polycrystal (textured)	293	19.0	710	2.0	[42]
Ni_44_Mn_46_Sn_10_	Polycrystal (textured)	320	18.0	390	3.0 × 10^−1^	[10]
Ni_45_Mn_44_Sn_11_	Polycrystal (textured)	298	10.0	310	3.0 × 10^−2^	[43]
Ni_47.5_Co_4.2_Mn_37.3_Sb_12.8_	Polycrystal (textured)	303	8.7	400	5.0 × 10^−2^	[44]
Cu_71.3_Al_17.5_Mn_11.2_	Single crystal	293	11.9	120	1.4	[45]
Cu_59.1_Zn_27_Al_13.8_Zr_0.1_	Single crystal	343	14.2	200	2.0 × 10^−1^	[46]
Ni_48.9_Ti_51.1_	Wire	322	21.0	900	2.0 × 10^−1^	[47]
Ni_50.8_Ti_49.2_	Polycrystal (textured)	323	17.9	913	2.0 × 10^−1^	[46]

**Table 2:** Cyclability and degradation rate of the elastocaloric effect for the present alloy and some elastocaloric materials.

Alloy	Sample Status	Number of Cycles	Degradation Rate of Δ*T_ad_* (K per Cycle)	Ref.
(Ni_50_Mn_31_Ti_19_)_99_B_1_	Polycrystal (textured)	12,000	7.5 × 10^−5^	This work
Ni_50_Mn_30_Ti_20_	Polycrystal (textured)	2000	5.0 × 10^−4^	[39]
Ni_37_Co_9_Fe_4_Mn_35_Ti_15_	Polycrystal (untextured)	1000	2.0 × 10^−4^	[57]
Ni_50.4_Mn_27.3_Ga_22.3_	Polycrystal (textured)	250	1.2 × 10^−3^	[66]
Ni_50.4_Mn_27.3_Ga_22.3_	Polycrystal (textured)	100	2.0 × 10^−3^	[12]
Ni_50_Fe_19_Ga_27_Co_4_	Single crystal	3000	9.3 × 10^−4^	[20]
Ni_54_Fe_19_Ga_27_	Single crystal	100	1.0 × 10^−3^	[67]
Ni_54_Fe_19_Ga_27_	Polycrystal (untextured)	100	9.0 × 10^−3^	[59]
Ni_53.2_Fe_19.4_Ga_27.4_	Polycrystal (untextured)	480	2.3 × 10^−3^	[60]
Cu_71.3_Al_17.5_Mn_11.2_	Single crystal	50	4.0 × 10^−3^	[45]
Cu_71_Al_18_Mn_11_	Polycrystal (textured)	275	1.5 × 10^−3^	[61]
Cu_71.1_Al_17.2_Mn_11.7_	Polycrystal (textured)	200	1.0 × 10^−3^	[53]
Cu_59.1_Zn_27_Al_13.8_	Polycrystal (untextured)	10,000	1.4 × 10^−4^	[46]
Co_49_Fe_3_V_33_Ga_15_	Polycrystal (textured)	200	1.5 × 10^−3^	[62]
Co_50_V_35_Ga_14_Ni_1_	Polycrystal (untextured)	4000	8.5 × 10^−4^	[63]
(Ni_42.5_Ti_50_Cu_7.5_)_99_Co_1_	Polycrystal (textured)	200	2.2 × 10^−2^	[64]
Ti_54.9_Ni_32.5_Cu_12.6_	Polycrystal (untextured)	1502	2.0 × 10^−4^	[65]

## Data Availability

Data will be made available on request.

## Supplementary Materials

The following supporting information can be downloaded at: https://www.mdpi.com/article/10.3390/ma17194756/s1, Figure S1: Compressive stress–strain curve for the arc-melted (Ni50Mn31Ti19)99B1 alloy measured at 293 K; Figure S2: EBSD orientation micrograph for the arc-melted (Ni50Mn31Ti19)99B1 alloy and corresponding inverse pole figure; Figure S3: Compressive stress–strain curves for the directionally solidified (Ni50Mn31Ti19)99B1 alloy during 10 cycles of superelastic training; Figure S4: Temperature dependence of the specific heat capacity Cp measured on heating for the directionally solidified alloy; Figure S5: Cyclic tests for the directionally solidified Ni_50_Mn_31_Ti_19_ alloy.

## References

[1] Lloveras P., Stern-Taulats E., Barrio M., Tamarit J.L., Crossley S., Li W., Pomjakushin V., Planes A., Mañosa L., Mathur N.D. (2015). Giant barocaloric effects at low pressure in ferrielectric ammonium sulphate. Nat. Commun..

[2] Mañosa L., Planes A., Acet M. (2013). Advanced materials for solid-state refrigeration. J. Mater. Chem. A.

[3] Tušek J., Engelbrecht K., Millán-Solsona R., Mañosa L., Vives E., Mikkelsen L.P., Pryds N. (2015). The elastocaloric effect: A way to cool efficiently. Adv. Energy Mater..

[4] Mañosa L., Planes A. (2017). Materials with giant mechanocaloric effects: Cooling by strength. Adv. Mater..

[5] Yang J., Wang H., Li Z., Zou N., Yan H., Yang B., Zuo L. (2024). Crystallography of stress-induced martensitic transformation and giant elastocaloric effect in a <001>_A_ textured Ni_27_Cu_21_Mn_46_Sn_6_ shape memory alloy. Acta Mater..

[6] Heczko O., Cejpek P., Drahokoupil J., Holý V. (2016). Structure and microstructure of Ni-Mn-Ga single crystal exhibiting magnetic shape memory effect analysed by high resolution X-ray diffraction. Acta Mater..

[7] Heczko O., Kopeček J., Straka L., Seiner H. (2013). Differently mobile twin boundaries and magnetic shape memory effect in 10M martensite of Ni–Mn–Ga. Mater. Res. Bull..

[8] Liu J., Gottschall T., Skokov K.P., Moore J.D., Gutfleisch O. (2012). Giant magnetocaloric effect driven by structural transitions. Nat. Mater..

[9] Gottschall T., Skokov K.P., Frincu B., Gutfleisch O. (2015). Large reversible magnetocaloric effect in Ni-Mn-In-Co. Appl. Phys. Lett..

[10] Zhang G.Y., Li Z.B., Yang J.J., Yang B., Wang D.H., Zhang Y.D., Esling C., Hou L., Li X., Zhao X. (2020). Giant elastocaloric effect in a Mn-rich Ni_44_Mn_46_Sn_10_ directionally solidified alloy. Appl. Phys. Lett..

[11] Wang H., Li D., Zhang G., Li Z., Yang B., Yan H., Cong D., Esling C., Zhao X., Zuo L. (2022). Highly sensitive elastocaloric response in a directionally solidified Ni_50_Mn_33_In_15.5_Cu_1.5_ alloy with strong <001>_A_ preferred orientation. Intermetallics.

[12] Wei L., Zhang X., Liu J., Geng L. (2018). Orientation dependent cyclic stability of the elastocaloric effect in textured Ni-Mn-Ga alloys. AIP Adv..

[13] Yang Z., Cong D.Y., Sun X.M., Nie Z.H., Wang Y.D. (2017). Enhanced cyclability of elastocaloric effect in boron-microalloyed Ni-Mn-In magnetic shape memory alloys. Acta Mater..

[14] Yan H.L., Wang L.D., Liu H.X., Huang X.M., Jia N., Li Z.B., Yang B., Zhang Y.D., Esling C., Zhao X. (2019). Giant elastocaloric effect and exceptional mechanical properties in an all-d-metal Ni–Mn–Ti alloy: Experimental and ab-initio studies. Mater. Des..

[15] Cong D.Y., Xiong W.X., Planes A., Ren Y., Manosa L., Cao P.Y., Nie Z.H., Sun X.M., Yang Z., Hong X.F. (2019). Colossal elastocaloric effect in ferroelastic Ni-Mn-Ti alloys. Phys. Rev. Lett..

[16] Li Y., Sun W., Zhao D., Xu H., Liu J. (2017). An 8 K elastocaloric temperature change induced by 1.3% transformation strain in Ni_44_Mn_45−x_Sn_11_Cu_x_ alloys. Scr. Mater..

[17] Ma G., Li C., Chen M., Zong S., Zhang Y., Zhao S., Chen F., Xuan H. (2022). Elastocaloric effect and magnetic properties of Ni_50_Mn_31.5_Ti_18_Cu_0.5_ shape memory alloy. J. Supercond. Novel Magn..

[18] Fornell J., Tuncer N., Schuh C.A. (2017). Orientation dependence in superelastic Cu-Al-Mn-Ni micropillars. J. Alloys Compd..

[19] Villa F., Villa E., Righi L., Ruggieri P., Bennato N., Battiston S., Passaretti F., Casati R. (2024). Effect of the thermal processing on the microstructural, functional and mechanical properties of cast polycrystalline NiMnTi alloys. J. Alloys Compd..

[20] Xiao F., Jin M.J., Liu J., Jin X.J. (2015). Elastocaloric effect in Ni_50_Fe_19_Ga_27_Co_4_ single crystals. Acta Mater..

[21] Fazeli S., Izadifar M., Dolado J.S., Ramazani A., Sadrnezhaad S.K. (2022). Atomistic study of the effect of crystallographic orientation on the twinning and detwinning behavior of NiTi shape memory alloys. Comput. Mater. Sci..

[22] Hsu Y.T., Wu C.T., Chen C.H. (2024). Nanoscale-precipitate-strengthened (Ni,Cu)-rich TiNiCu shape memory alloy with stable superelasticity and elastocaloric performance. J. Alloys Compd..

[23] Meng J., Xie L., Yu Q., Wang J., Jiang C. (2024). Toughening the grain boundaries by introducing a small amount of the second phase: Ni-Cu-Mn-Ga shape memory alloys as an example. Acta Mater..

[24] Guan Z., Bai J., Zhang Y., Sun S., Gu J., Liang X., Zhang Y., Esling C., Zhao X., Zuo L. (2023). Achieved good mechanical properties and large elastocaloric effect in Ni-Mn-Ti-Cu-B alloy: Experiments and first-principles calculations. J. Alloys Compd..

[25] Zhang Y., Yang S., Wang L., Pan S., Zhang J., Liu X., Wang C. (2023). Development of boron-microalloyed Co–V–Al–Fe shape memory alloys. Intermetallics.

[26] Li X., Fautrelle Y., Gagnoud A., Du D., Wang J., Ren Z., Nguyen-Thi H., Mangelinck-Noel N. (2014). Effect of a weak transverse magnetic field on solidification structure during directional solidification. Acta Mater..

[27] Li B., Li S.M., Yang B., Zhong H., Liu Z.P., Li D. (2023). Enhancing the elastocaloric effect and thermal cycling stability in dendritic-like Ni_50_Mn_31.6_Ti_18.4_ single crystal. J. Alloys Compd..

[28] Xu F., Zhu C., Wang J., Luo F., Zhu X., Xu J., Chen S., Wang J., Ma G., Chen F. (2023). Enhanced elastocaloric effect and mechanical properties of Gd-doped Ni-Co-Mn-Ti-Gd metamagnetic shape memory alloys. J. Alloys Compd..

[29] Li B., Zheng L.J., Zhang H. (2024). Microstructure-property relationship in Zr-alloyed Ni-rich NiTi alloys: Enhancements in high-temperature stability and superelasticity. Mater. Charact..

[30] Zhao Y., Hou L., Li X., Su H., Zhang J. (2024). On the formation of gradient-distributed dendrites in a single crystal nickel-based superalloy directionally solidified under transverse static magnetic field. Mater. Charact..

[31] Niu Y., Chen H., Zhang X., Li S., Cong D., Ma T., Li S., Lin J., Wang Y.D. (2021). Achieving excellent superelasticity and extraordinary elastocaloric effect in a directionally solidified Co-V-Ga alloy. Scr. Mater..

[32] Zhao Y., Ming F., Jia N., Chen J., Ren S., Xu W., Qu X. (2021). High-strength superelastic as-cast Ni_50.9_Ti_49.1_-TiB_2_ in-situ composites. Mater. Sci. Eng. A.

[33] Pfeuffer L., Lemke J., Shayanfar N., Riegg S., Koch D., Taubel A., Scheibel F., Kani N.A., Adabifiroozjaei E., Molina-Luna L. (2021). Microstructure engineering of metamagnetic Ni-Mn-based Heusler compounds by Fe-doping: A roadmap towards excellent cyclic stability combined with large elastocaloric and magnetocaloric effects. Acta Mater..

[34] Zhang X., Chen H., Li S., Niu Y., Yin T., Song C., Lang R., Cong D., Li S., Wang Y.D. (2022). Enhanced cyclability of superelasticity and elastocaloric effect in Cu and B co-doped Co-Ni-Ga shape memory alloys. J. Alloys Compd..

[35] Zhang X., Zhang M., Cui T., Li J., Liu Q., Wang H. (2019). The enhancement of the mechanical properties and the shape memory effect for the Cu-13.0Al-4.0Ni alloy by boron addition. J. Alloys Compd..

[36] Li X.W., Yang M.H., Sun C.H., Ruan Y., Wei B. (2024). Enhanced tensile performance of ternary Fe-Ni-Ti peri-eutectic alloy through directional solidification associated with thermal processing. Mater. Lett..

[37] Qian S.X., Ling J.Z., Hwang Y., Radermacher R., Takeuchi I. (2015). Thermodynamics cycle analysis and numerical modeling of thermoelastic cooling systems. Int. J. Refrig..

[38] Moya X., Defay E., Heine V., Mathur N.D. (2015). Too cool to work. Nat. Phys..

[39] Zhang G., Wang H., Li Z., Yang B., Yan H., Zuo L. (2023). Giant elastocaloric effect covering a wide temperature region in a directionally solidified Ni_50_Mn_30_Ti_20_ alloy. Scr. Mater..

[40] Li D., Li Z., Yang J., Li Z., Yang B., Yan H., Wang D., Hou L., Li X., Zhang Y. (2019). Large elastocaloric effect driven by stress-induced two-step structural transformation in a directionally solidified Ni_55_Mn_18_Ga_27_ alloy. Scr. Mater..

[41] Zhao D., Castán T., Planes A., Li Z., Sun W., Liu J. (2017). Enhanced caloric effect induced by magnetoelastic coupling in NiMnGaCu Heusler alloys: Experimental study and theoretical analysis. Phys. Rev. B.

[42] Huang X.M., Zhao Y., Yan H.L., Tang S., Yang Y., Jia N., Yang B., Li Z., Zhang Y., Esling C. (2023). A first-principle assisted framework for designing high elastocaloric Ni–Mn-based magnetic shape memory alloy. J. Mater. Sci. Technol..

[43] Sun W., Liu J., Zhao D., Zhang M. (2017). Directional solidification and elastocaloric effect in a Ni_45_Mn_44_Sn_11_ magnetic shape memory alloy. J. Phys. D Appl. Phys..

[44] Li Z., Li Z., Yang J., Li D., Yang B., Yan H., Nie Z., Hou L., Li X., Zhang Y. (2019). Large elastocaloric effect in a polycrystalline Ni_45.7_Co_4.2_Mn_37.3_Sb_12.8_ alloy with low transformation strain. Scr. Mater..

[45] Wang Y., Liu C., Wang H., Li Z., Li J., Yang B., Yan H., Zuo L. (2023). Orientation dependence of elastocaloric effect in a Cu_71.3_Al_17.5_Mn_11.2_ single crystal. J. Alloys Compd..

[46] Wu Y., Ertekin E., Sehitoglu H. (2017). Elastocaloric cooling capacity of shape memory alloys—Role of deformation temperatures, mechanical cycling, stress hysteresis and inhomogeneity of transformation. Acta Mater..

[47] Tušek J., Engelbrecht K., Mikkelsen L.P., Pryds N. (2015). Elastocaloric effect of Ni-Ti wire for application in a cooling device. J. Appl. Phys..

[48] Qian S.X., Geng Y.L., Wang Y., Muehlbauer J., Ling J.Z., Hwang Y., Radermacher R., Takeuchi I. (2016). Design of a hydraulically driven compressive elastocaloric cooling system. Sci. Technol. Built Environ..

[49] Wang J., Yu Q., Xu K., Zhang C., Wu Y., Jiang C. (2017). Large room-temperature elastocaloric effect of Ni_57_Mn_18_Ga_21_In_4_ alloy undergoing a magnetostructural coupling transition. Scr. Mater..

[50] Masdeu F., Pons J., Torrens-Serra J., Chumlyakov Y., Cesari E. (2022). Superelastic behavior and elastocaloric effect in a Ni_51.5_Fe_21.5_Ga_27.0_ ferromagnetic shape memory single crystal under compression. Mater. Sci. Eng. A.

[51] Li Z.Z., Li Z.B., Li D., Yang J.J., Yang B., Hu Y., Wang D.H., Zhang Y.D., Esling C., Zhao X. (2020). Achieving a broad refrigeration temperature region through the combination of successive caloric effects in a multiferroic Ni_50_Mn_35_In_15_ alloy. Acta Mater..

[52] Zhao D.W., Liu J., Chen X., Sun W., Li Y., Zhang M.X., Shao Y.Y., Zhang H., Yan A. (2017). Giant caloric effect of low-hysteresis metamagnetic shape memory alloys with exceptional cyclic functionality. Acta Mater..

[53] Yuan B., Zhong S., Qian M., Zhang X., Geng L. (2021). Elastocaloric effect in bamboo-grained Cu_71.1_Al_17.2_Mn_11.7_ microwires. J. Alloys Compd..

[54] Xu S., Huang H., Xie J., Takekawa S., Xu X., Omori T., Kainuma R. (2016). Giant elastocaloric effect covering wide temperature range in columnar-grained Cu_71.5_Al_17.5_Mn_11_ shape memory alloy. APL Mater..

[55] Liu C., Li Z.B., Wang H.L., Wang Y.P., Yang B., Yan H.L., Cong D.Y., Zhao X., Zuo L. (2022). Long-term stable elastocaloric effect in a Heusler-type Co_51_V_33_Ga_16_ polycrystalline alloy. ACS Appl. Energy Mater..

[56] Zhou M., Li Y., Zhang C., Li L. (2018). Elastocaloric effect and mechanical behavior for NiTi shape memory alloys. Chin. Phys. B.

[57] Liu S., Xuan H., Cao T., Wang L., Xie Z., Liang X., Li H., Feng L., Chen F., Han P. (2019). Magnetocaloric and elastocaloric effects in all-d-metal Ni_37_Co_9_Fe_4_Mn_35_Ti_15_ magnetic shape memory alloy. Phys. Status Solidi.

[58] Sidharth R., Abuzaid W., Vollmer M., Niendorf T., Sehitoglu H. (2020). Fatigue crack initiation in the iron-based shape memory alloy FeMnAlNiTi. Shape Mem. Superelasticity.

[59] Xu Y., Lu B., Sun W., Yan A., Liu J. (2015). Large and reversible elastocaloric effect in dual-phase Ni_54_Fe_19_Ga_27_ superelastic alloys. Appl. Phys. Lett..

[60] Imran M., Zhang X., Qian M., Geng L. (2021). Enhanced working stability of elastocaloric effects in polycrystalline Ni-Fe-Ga dual phase alloy. Intermetallics.

[61] Yuan B., Qian M., Zhang X., Imran M., Geng L. (2020). Enhanced cyclic stability of elastocaloric effect in oligocrystalline Cu–Al–Mn microwires via cold-drawing. Int. J. Refrig.

[62] Liu K., Yuan Y., Ma S., Feng G., Wan D., Wang S., Chen C., Luo X., Zhong Z. (2021). Large elastocaloric effect around room temperature in directionally solidified Co_49_Fe_3_V_33_Ga_15_ superelastic alloy. J. Alloys Compd..

[63] Liu C., Li D., Li Z., Yang B., Yan H., Li J., Li Z., Zhao X., Zuo L. (2021). Large elastocaloric effect in a Heusler-type Co_50_V_35_Ga_14_Ni_1_ polycrystalline alloy. Appl. Phys. Lett..

[64] Yang Z., Cong D., Yuan Y., Li R., Zheng H., Sun X., Nie Z., Ren Y., Wang Y. (2020). Large room-temperature elastocaloric effect in a bulk polycrystalline Ni-Ti-Cu-Co alloy with low isothermal stress hysteresis. Appl. Mater. Today.

[65] Bechtold C., Chluba C., Lima de Miranda R., Quandt E. (2012). High cyclic stability of the elastocaloric effect in sputtered TiNiCu shape memory films. Appl. Phys. Lett..

[66] Wei L., Zhang X., Gan W., Ding C., Geng L. (2019). Hot extrusion approach to enhance the cyclic stability of elastocaloric effect in polycrystalline Ni-Mn-Ga alloys. Scr. Mater..

[67] Eftifeeva A., Panchenko E., Yanushonite E., Kurlevskaya I., Timofeeva E., Tokhmetova A., Surikov N., Tagiltsev A., Chumlyakov Y. (2022). Superelasticity and elastocaloric cooling capacity in stress-induced martensite aged [001]_А_-oriented Ni_54_Fe_19_Ga_27_ single crystals. Mater. Sci. Eng. A.

[68] Liang X., Xiao F., Jin X., Fukuda T., Kakeshita T. (2017). Suppression of martensitic transformation in Co_2_Cr(Ga,Si) Heusler alloys by thermal cycling. Metall. Mater. Trans. A.

[69] Sidharth R., Celebi T.B., Sehitoglu H. (2024). Origins of functional fatigue and reversible transformation of precipitates in NiTi shape memory alloy. Acta Mater..

[70] Dadda J., Maier H.J., Niklasch D., Karaman I., Karaca H.E., Chumlyakov Y.I. (2008). Pseudoelasticity and cyclic stability in Co_49_Ni_21_Ga_30_ shape-memory alloy single crystals at ambient temperature. Metall. Mater. Trans. A.

[71] Fu H., Xu S., Zhao H., Dong H., Xie J. (2017). Cyclic stress-strain response of directionally solidified polycrystalline Cu-Al-Ni shape memory alloys. J. Alloys Compd..

[72] Briant C.L., Messmer R.P. (1980). Electronic effects of sulphur in nickel. Philos. Mag. B.

[73] Messmer R.P., Briant C.L. (1982). The role of chemical bonding in grain boundary embrittlement. Acta Metall..

[74] Schulson E.M., Weihs T.P., Baker I., Frost H.J., Horton J.A. (1986). Grain boundary accommodation of slip in Ni_3_Al containing boron. Acta Metall..

[75] Liu Y., Liu C.T., Heatherly L., George E.P. (2011). Effect of boron on the fracture behavior and grain boundary chemistry of Ni_3_Fe. Scr. Mater..

[76] Liu C.T., George E.P. (1990). Environmental embrittlement in boron-free and boron-doped FeAl (40 at. % Al) alloys. Scr. Metall. Mater..

