# Supplementary material for: Giant Elastocaloric Effect and Improved Cyclic Stability in a Directionally Solidified (Ni50Mn31Ti19)99B1 Alloy
Source: Materials (Basel). 2024 Sep 27;17(19):4756. doi: 10.3390/ma17194756 (PMC11477505; doi:10.3390/ma17194756)
Supplement: Supplementary file 1 [file materials-17-04756-s001.zip › materials-3167742-supplementary.pdf]

Supporting Information

Giant elastocaloric effect and long-term cyclic stability in a directionally solidified $(Ni_{50}Mn_{31}Ti_{19})_{99}B_1$ alloy

Honglin Wang[1], Yueping Wang[1], Guoyao Zhang[1], Zongbin Li[1,*], Jiajing Yang[2], Jinwei Li[3], Bo Yang[1], Haile Yan[1], Liang Zuo[1]

[1]Key Laboratory for Anisotropy and Texture of Materials (Ministry of Education), School of Materials Science and Engineering, Northeastern University, Shenyang 110819, People's Republic of China.

[2]Western Metal Materials Co., Ltd, Xi'an 710201, People's Republic of China.

[3]Liaoning Automobile Lightweight Professional Technology Innovation Center, Tieling 112000, People's Republic of China.

**Corresponding authors. E-mail: lizb@atm.neu.edu.cn.*

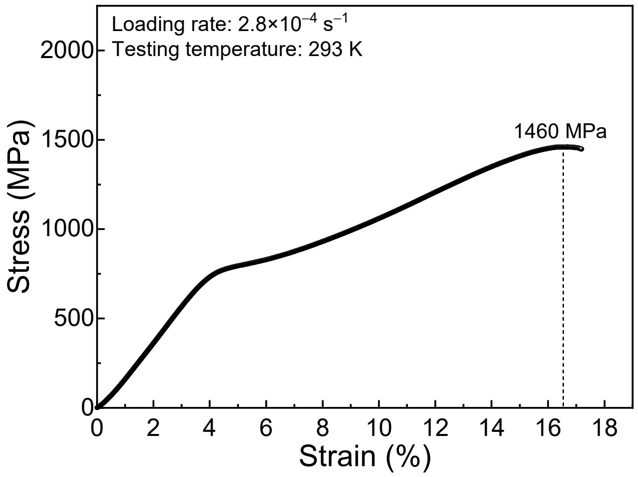

Figure S1. Compressive stress-strain curve for the arc-melted $(Ni_{50}Mn_{31}Ti_{19})_{99}B_1$ alloy measured at 293 K.

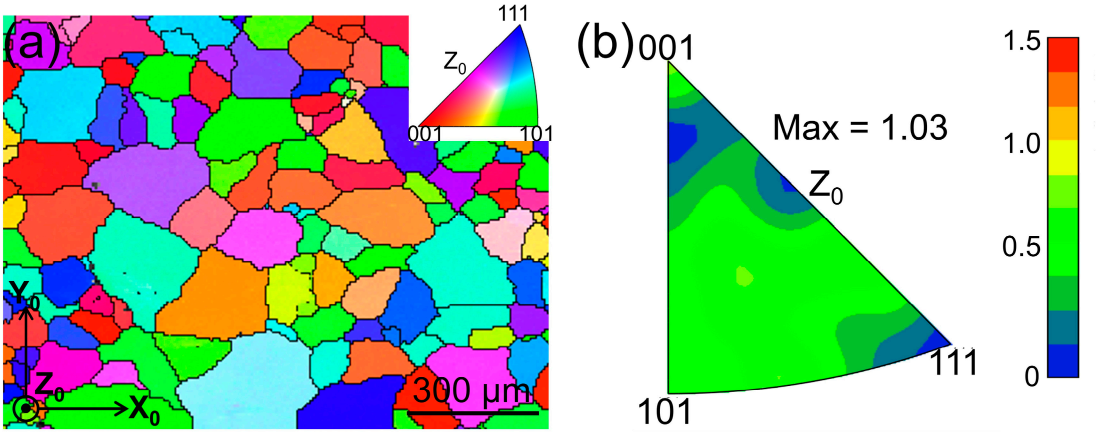

Figure S2. (**a**) EBSD orientation micrograph (IPF contrast) for the arc-melted $(Ni_{50}Mn_{31}Ti_{19})_{99}B_1$ alloy. (**b**) Corresponding inverse pole figure referring to the Z_0 axis.

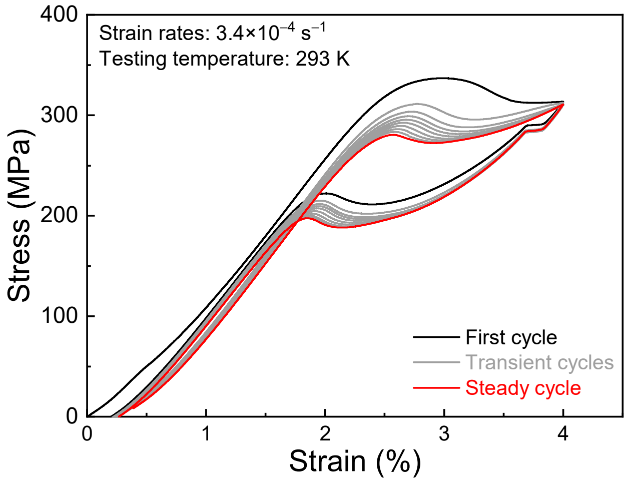

Figure S3. Compressive stress-strain curves for the directionally solidified $(Ni_{50}Mn_{31}Ti_{19})_{99}B_1$ alloy during 10 cycles of superelastic training.

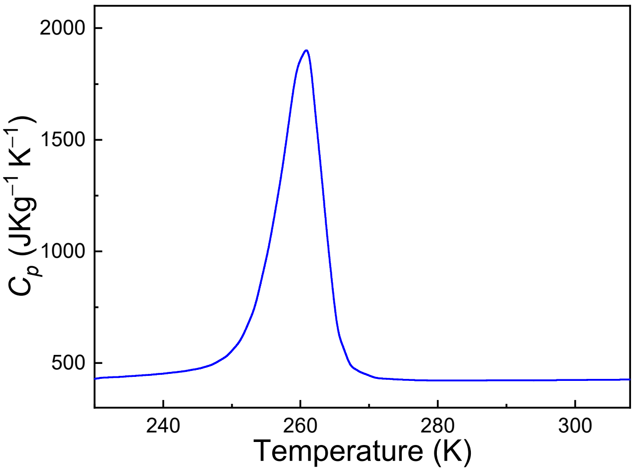

Figure S4. Temperature dependence of the specific heat capacity C_p measured on heating for the directionally solidified alloy.

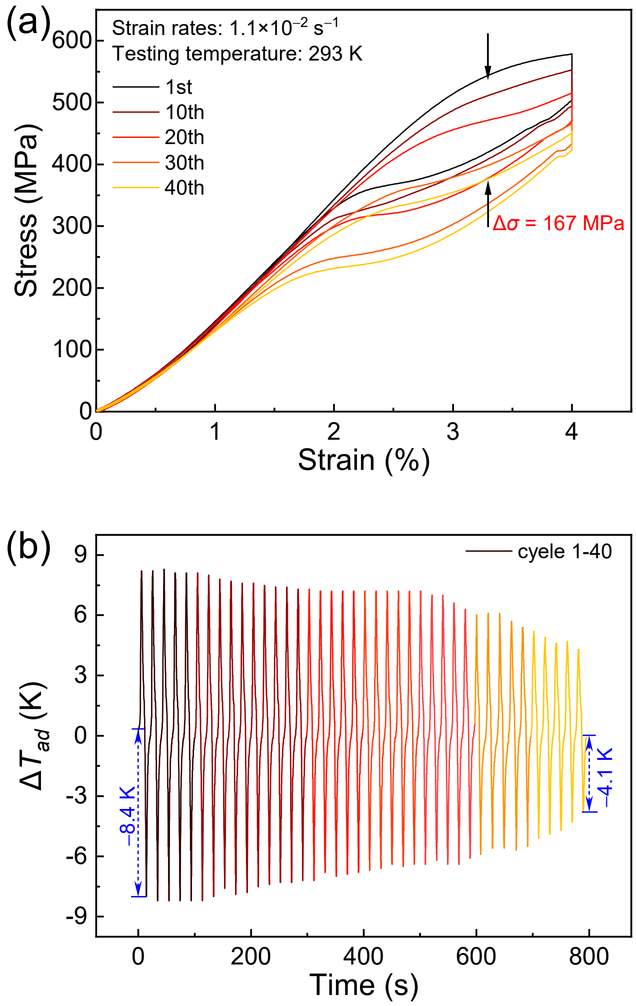

Figure S5. (**a**) Cyclic strain-stress curves for the directionally solidified $Ni_{50}Mn_{31}Ti_{19}$ alloy under the compressive strain of 4% at 293 K with the strain rates of 1.1 s^{-1}; (**b**) ΔT_{ad} profiles during cyclic loading/unloading tests.
